# Clinical challenge: fatal mucormycotic osteomyelitis caused by Rhizopus microsporus despite aggressive multimodal treatment

**DOI:** 10.1186/1471-2334-14-488

**Published:** 2014-09-06

**Authors:** Norbert Harrasser, Ingo J Banke, Matthias Hauschild, Ulrich Lenze, Peter M Prodinger, Andreas Toepfer, Christian Peschel, Rüdiger von Eisenhart-Rothe, Ingo Ringshausen, Mareike Verbeek

**Affiliations:** Clinic of Orthopedics and Sports Orthopedics, Klinikum rechts der Isar, Technical University of Munich, Ismaninger Str. 22, 81675 Munich, Germany; III. Department of Internal Medicine Klinikum rechts der Isar, Technical University of Munich, Ismaninger Str. 22, 81675 Munich, Germany

**Keywords:** Mucormycosis, Rhizopus microsporus, Fungal osteomyelitis, Immunocompromitation, Radical surgical proximal femoral resection

## Abstract

**Background:**

Mucormycosis is an invasive mycotic disease caused by fungi in the zygomycetes class. Although ubiquitous in the environment, zygomycetes are rarely known to cause invasive disease in immunocompromised hosts with a high mortality even under aggressive antifungal and surgical therapy. Clinically, mucormycosis frequently affects the sinus occasionally showing pulmonary or cerebral involvement. However skeletal manifestation with Rhizopus microsporus (RM) osteomyelitis leading to emergency surgical proximal femoral resection with fatal outcome has not been described yet.

**Case presentation:**

We report the case of a 73-year-old male suffering from myelodysplastic syndrome with precedent bone marrow transplantation. Six months after transplantation he consulted our internal medicine department in a septic condition with a four week history of painful swelling of the right hip. Radiography, computed tomography and magnetic resonance imaging revealed multiple bone infarcts in both femurs. In the right femoral head, neck and trochanteric region a recent infarct showed massive secondary osteomyelitis, breaking through the medial cortex. Emergency surgical proximal femoral resection was performed due to extensive bone and soft tissue destruction. Microbiological and basic local alignment search tool (BLAST) analysis revealed RM. Amphotericin B and posaconazole treatment with septic revision surgery was performed. However the disease ran a rapid course and was fatal two months after hospital admission.

**Conclusion:**

This alarming result with extensive RM osteomyelitis in the proximal femur of an immunocompromised patient may hopefully warn medical staff to perform early imaging and aggressive surgical supported multimodal treatment in similar cases.

**Electronic supplementary material:**

The online version of this article (doi:10.1186/1471-2334-14-488) contains supplementary material, which is available to authorized users.

## Background

Zygomycetes are environmental nonseptate molds widely distributed in soil, plants, and decaying material [1, 2]. The class zygomycetes contain the order mucorales, the latter including the genus Rhizopus. A clinically important Rhizopus species is Rhizopus microsporus (RM), being one of the main causes of mucormycosis, an opportunistic life-threatening infection in immunocompromised patients. Fungi like Rhizopus, Mucor and Rhizomucor account for over 75% of all mucormycosis cases [3–5]. Being ubiquitous in the environment, acquirement is usually generated through inhalation of spores often leading to rhinocerebral (39%) or pulmonary (24%), seldom to cutaneous (19%) or disseminated disease (23%) in predisposed individuals [2, 6, 7].

Previously reported risk factors for mucormycosis are prolonged neutropenia, immunosuppression, iron overload and prolonged hyperglycemia or manifest diabetes. Patients treated with allogeneic hematopoietic stem cell transplantation (allo-HSCT) often suffer from a combination of these risk factors [1]. The prognosis and outcome of invasive mucormycosis in patients with immune deficiency or hematologic malignancies is generally rather poor [1, 4, 8]. However in most reported cases fatal outcome could be prevented [8, 9].

Here we describe the unique case of fatal invasive osteomyelitis in an allo-HSCT recipient caused by RM. Extensive diagnostic evaluation revealed multiple old bone infarcts complicated with invasive fungal disease. Although systemic antifungal treatment and repetitive radical surgery was started immediately cure could not be provided.

## Case presentation

We report on a 73-year-old male with recently diagnosed myelodysplastic syndrome RAEB I showing complex karyotype. A routine-checkup revealed a tricytopenia in the blood count as well as a mild splenomegaly, further examinations including bone marrow biopsy confirmed the diagnosis. Facing the high-risk constellation of this disease (IPSS-R risk score: poor; [10]) and the excellent clinical condition of the patient with no relevant comorbidities, allogeneic stem cell transplantation was considered the sole option for a cure. With no HLA-identical siblings available, unrelated donor search was initiated resulting in the identification of a suitable HLA-matched donor.

Eleven months later allogeneic matched unrelated donor stem cell transplantation after conditioning chemotherapy with fludarabine, treosulfane as well as antithymocyte globuline and prophylactic immunosuppressive medication containing of cyclosporine and mycophenolatmofetile was performed successfully. No relevant complications occurred during the first weeks of follow-up besides moderate acute graft versus host disease of the skin which was immediately responsive to steroid treatment. Bone marrow examinations one month after transplantation showed complete cytogenetic remission of the disease as well as complete donor chimerism. With no further signs of graft versus host disease and a good clinical condition of the patient the immunosuppressive treatment could be constantly tapered during the following months. Continuous prophylactic antiinfectious medication with acyclovir, co-trimoxazole and posaconazole was administered.

Six months after transplantation without remaining medication the patient was presented to our internal emergency department with a critical septic condition showing fever (body temperature above 40°C) and dyspnea, and was immediately transferred to the intensive care unit (ICU). Intubation and mechanical ventilation had to be initiated due to respiratory failure. Computed tomography (CT) scan revealed bilateral infiltrations referring to atypical pneumonia and regional osteopaenia with mild focal bone lysis at the level of the lumbar spine. Lumbar spondylodiscitis was ruled out by magnetic resonance tomography imaging (MRI).

**Figure 1:**
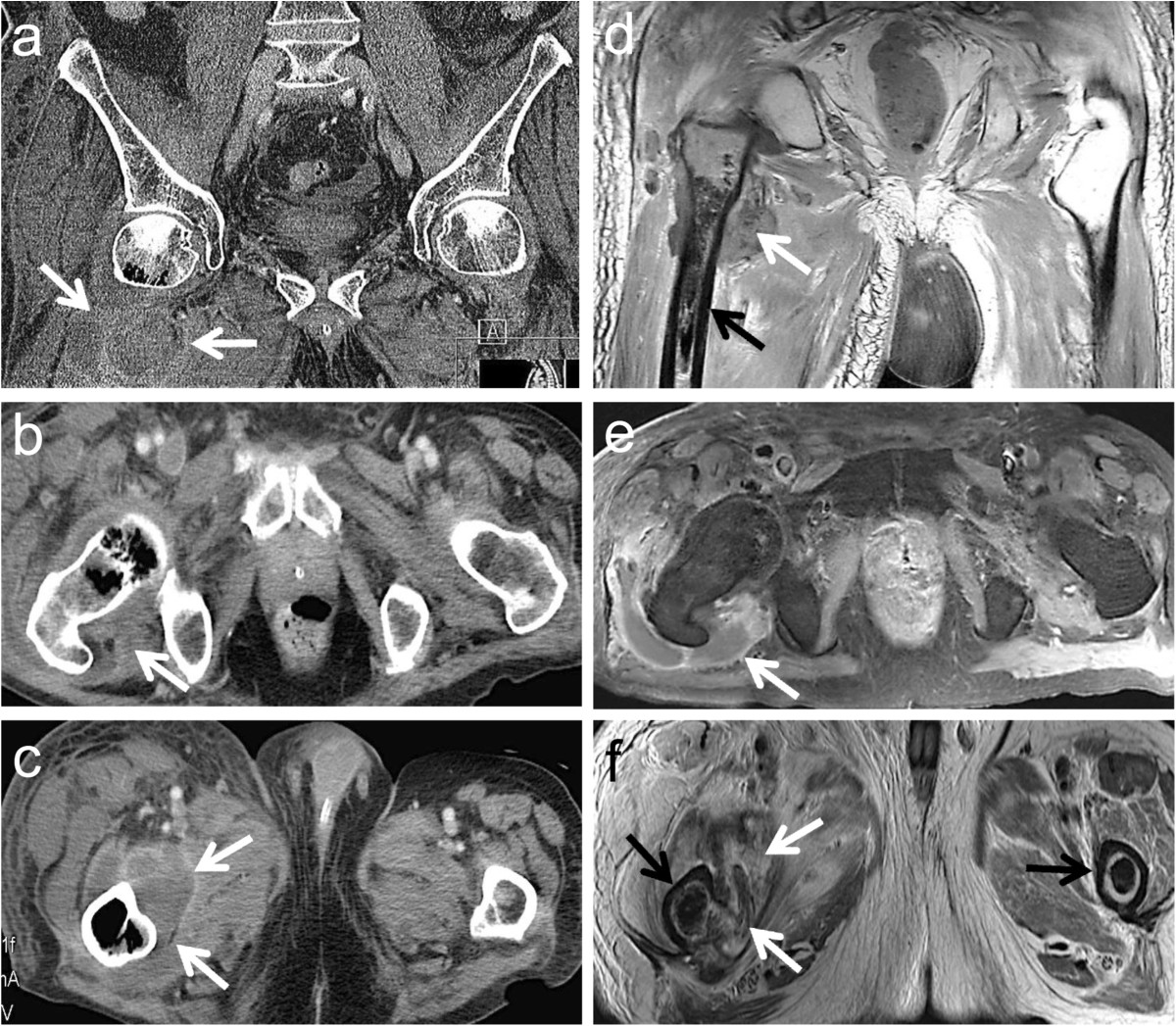
**Radiological assessment of the right hip showing large intramedullary cystic lesions with soft tissue abscesses.** Multipanel with coronal **(a, d)**, proximal transverse (femoral neck; **b, e**) and distal transverse (lesser trochanter; **c, f**) CT **(a, b, c)** and T2-weighted MRI **(d, e, f)** views focusing the right hip region. Large cavitary lesions with invasion of soft tissue corresponding to abscess formation (white arrows). Underlying bone marrow osteonecrosis of both femurs (black arrows).

**Figure 2:**
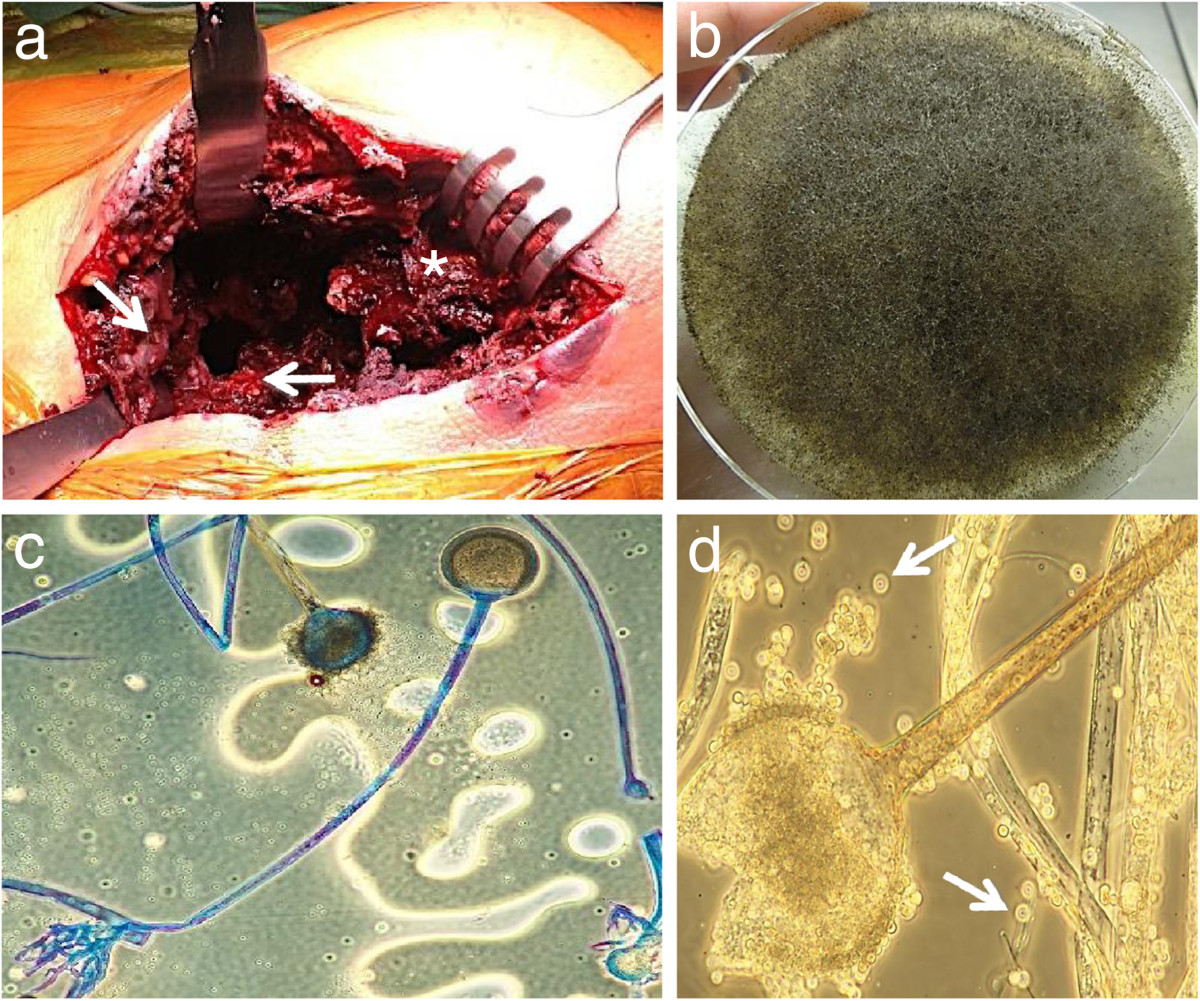
**Intraoperative aspect of the right hip demonstrating excessive destruction of bone and soft tissue with ubiquitous blackened tissue (a).** Femoral end (asterisk) after proximal femur resection with surrounding necrotic musculature (arrows). Culture specimen after 48 hours (40°C) showing grayish, fluffy RM colonies filling out the whole Sabouraud dextrose agar plate **(b)**. RM under 300-400× magnification **(c, d)**. Sporangia filled with sporangiospores (arrows) being partially extruded.

No relevant bacterial, viral or fungal cause could be identified by bronchoalveolar lavage and multiple blood culture collections during the stay on the ICU. Cardiac echocardiography was performed and culture-negative endocarditis could be ruled out. The patient constantly improved under combinatory empiric antibiotic, antiviral and antifungal (azole) medication, extubation could be performed 7 days after intubation. While the respiratory situation completely stabilized, the clinical condition of the patient constantly deteriorated in the following days. For the first time the patient reported right hip pain.In consequence ultrasound, CT and MRI of the right hip and thigh were performed in order to identify and localize a potential inflammatory focus. The imaging revealed multiple old bone infarcts in both femurs as well as a new infarct with massive secondary osteomyelitis in the right femoral head, neck and trochanteric region, breaking through the medial cortex into the surrounding soft tissue (Figure 1). With emergency surgical intervention extensive bone and soft tissue destruction with ubiquitous blackened tissue became evident (Figure 2a). Proximal femoral resection with a broad antibiotic (vancomycin, gentamycin, clindamycin) and antimycotic (amphotericin B) loaded spaceholder and extensive tissue debridement were performed. Microbiological and histopathological analysis and basic local alignment search tool (BLAST) identified RM (Figure 2b-d). Bacteria could not be identified even after prolonged microbiological culturing for 10 days. Immediate high dosed liposomal amphotericin B (6 mg/kg bodyweight/day), supplemented with high dosed posaconazole (4 × 200 mg/day) was administered. After a short-term improvement of the patient’s condition with promising regression of inflammatory markers and a fever-free period, the wound at the level of the proximal thigh showed increased wound secretion and shading of the surrounding skin. Despite another 3 surgical interventions with debridement, lavage and vacuum assisted closure-therapy (VAC) the intraoperative and cutaneous state deteriorated with RM invading the tissue in a diffuse manner and ultimately perforating the skin. With this dramatic disease progression a whole-body-MRI was conducted 6 weeks after re-admission. Multiple other bone infarctions (left femur, both humeri, both tibiae) with suspected superinfection of RM became evident. Considering the drastic disease progression under maximal multimodal care further therapeutic interventions were stopped in consent with the patient and his family and palliative home care support was initiated. The patient passed away a few days later.

## Discussion

Invasive disseminated Rhizopus-infections develop in fewer than ¼ of localized forms and have an estimated mortality rate of 78–100% in allo-HSCT recipients [8]. We describe the fatally ending case of a disseminated RM osteomyelitis of an immunocompromised patient suffering from myelodysplastic syndrome. To the best of our knowledge, a comparable case has not been reported in the literature yet.

Immunocompromised patients, particularly suffering from hematological diseases treated by allo-HSCT face a high risk of invasive fungal infections [6]. Invasive aspergillosis and candidiasis represent the leading cause of invasive mold infection, whereas invasive mucormycosis is less common [4]. A recent increase in the incidence of mucormycosis may be explained by the increasing use of antifungal agents lacking activity against the class zygomycetes and the growing number of high risk bone marrow transplantations [5]. Zygomycetes are found worldwide with distribution throughout the whole environment (e.g., air, soil, food, and wood). The class zygomycetes contain the order mucorales and the genus Rhizopus. Rhizopus can be further subdivided in several species. Some species e.g. RM are known human pathogens [6]. However, reports on RM-related infections are relatively rare compared with those related to other Rhizopus species [11]. Wilkins reported the case of a non-immunocompromised patient with a postoperative RM osteomyelitis of the femur after anterior cruciate ligament repair. Multimodal treatment led to eradication of the disease [12]. A successful treatment of RM osteomyelitis of the right tibia in an immunocompromised patient was presented by Vashi [9].

Compared to focal RM fungal infections disseminated infections are even more seldom [2, 13, 14]. Especially HSCT recipients run high risk of developing invasive fungal infections during the immediate post-transplant and the pre- and post-engraftment period and the engraftment period up to 3 months after transplantation [2]. Our patient showed suspected infection about 3 months after transplantation.

Mucormycosis spreads from isolated infection hematogenously to other organs. The most common sites of origin are sinuses (39%), lungs (24%), and skin (19%) [2, 15]. Dissemination commonly affects lung and brain, whereas liver, heart, and kidneys are rarely colonized [5]. Our patient showed multiple lesions in the final state as demonstrated by MRI. Due to the concomitant presence of bone necrosis and metastatic fungal implants we believe that initially bone infarction, caused by immonostatic drugs, lead to an ideal environment for fungal growth. A similar clinical constellation with Rhizopus species-superinfection after B-19 virus-induced bone marrow necrosis in a patient with sickle-cell disease was presented by Fartoukh et al. [16]. However, surgical intervention was not performed. In our case the focus of verifiable RM infection was the right proximal femur and its surrounding soft tissues with huge abscess formations. Other lesions at the left lower extremity and both upper extremities obviously followed or had preexisted in a dormant state.

Furthermore establishing the diagnosis of invasive fungal infections, primarily based on standard culture-based mycological methods is often difficult, especially in early stages [6]. Accordingly high rates of delayed treatment beginnings are described [11]. Routinely taken blood culture samples confirm the diagnosis in less than 10% of all cases [17]. Therefore surgical intervention is often required not just for treatment but to gain tissue specimens to increase diagnostic accuracy. Direct microscopy with optical brighteners, microbial cultures and histopathology are recommended allowing a rapid narrowing-down of the diagnosis [18]. With direct microscopy hyphae of mucorales display a typical appearance. They show a variable width (6–25 μm), are non- or pauci-septate, have an irregular, ribbon-like appearance and a variable angle of branching. Differentiation between various genera is based on the presence and location of rhizoids, the branching nature of the sporangiophores, the shape of the columella, the size and shape of the sporangia, and the maximum growth temperature [19]. In addition to microscopy culture of specimens is considered an essential investigation. Although the sensitivity of culture is not high, it allows identification and susceptibility testing [18]. Currently molecular testing displays the most reliable diagnostic tool for identification of human pathogenic mucorales. As the detection of mucorales-specific antigens so far has not become generally accepted for diagnostic purposes because of its relatively low sensitivity [20], currently the most effective method for mucorales-detection is PCR [18]. The internal transcribed fungal spacer (ITS) region (18S rRNA and 28S rRNA) is sequenced and the isolates are identified by e.g. Basic Local Alignment Search Tool (BLAST®). In our case we were able to identify RM by a combination of phenotypic methods (Figure 2b-d) and genetic sequencing.

Therapy for mucormycosis infections includes systemic antifungal drugs and local surgical debridement [21]. Regarding antifungal treatment azole, which are used for aspergillosis are not appropriate for invasive mucormycosis. Moreover, mucormycosis can even arise in patients receiving azole for prophylaxis or for treatment of invasive aspergillosis [22, 23]. Being aware of our patient’s critical illness and according to current treatment concepts, systemic treatment was started with high-dosage liposomal amphotericin B and posaconazole after confirmation of the diagnosis [18, 24, 25]. Possibly due to this regimen, blood inflammatory markers temporarily decreased significantly and a fever-free interval was achieved. Amphotericin B, commonly accepted as first-line treatment for invasive mucormycosis can be combined with posaconazole, which is strongly recommended for salvage treatment [18]. After a short improvement of clinical status under systemic antifungal therapy the patient’s condition deteriorated again. Salvage surgery was indicated after MRI-proved progression of disease at the level of the right hip. Unfortunately the local situation was out of control at that time and further fungus invasion could not be stopped. Mortality rates of 10% have been reported for localized cutaneous mucormycosis, 26% after extension to deeper structures, and 94% with disseminated disease [8]. These rates clearly emphasize the importance of early aggressive therapeutic intervention. In our patient early dissemination was present lowering chances of cure significantly from the beginning.

## Conclusion

This dramatic case of rapidly progressive and ultimately fatal RM infection of the bone illustrates the diagnostic and therapeutic challenges of mucormycosis in immunocompromised hosts. Amongst others, abscess formations should always be suspicious of invasive fungal infection under these circumstances. Rapid and exact diagnosis by both morphology and molecular techniques is crucial for starting early treatment of fungal infection. Amphotericine B should be used as soon as mucormycosis is suspected. If the patient’s condition is not improving, addition of posaconazole should be considered. At the same time, early and aggressive surgical debridement has to be performed first to detect the pathogen and second to establish local control. Comprehensive multimodal therapy for mucormycosis may create more opportunities to improve patient’s outcome despite the very low overall survival rate in disseminated cases.

## Consent

Written informed consent was obtained from the patient for publication of this case report and any accompanying images. A copy of the written consent is available for review by the editor of this journal.

## Authors’ original submitted files for images

Below are the links to the authors’ original submitted files for images. Authors’ original file for figure 1 Authors’ original file for figure 2

## Abbreviations

allo-HSCT: Allogeneic hematopoietic stem cell transplantation
BLAST: Basic local alignment search tool
CT: Computed tomography
ICU: Intensive care unit
MRI: Magnetic resonance imaging
RM: Rhizopus microsporus.
