# Supplementary figures and images for: Clinical challenge: fatal mucormycotic osteomyelitis caused by Rhizopus microsporus despite aggressive multimodal treatment
Source: BMC Infect Dis. 2014 Sep 6;14:488. doi: 10.1186/1471-2334-14-488 (PMC4164739; doi:10.1186/1471-2334-14-488)

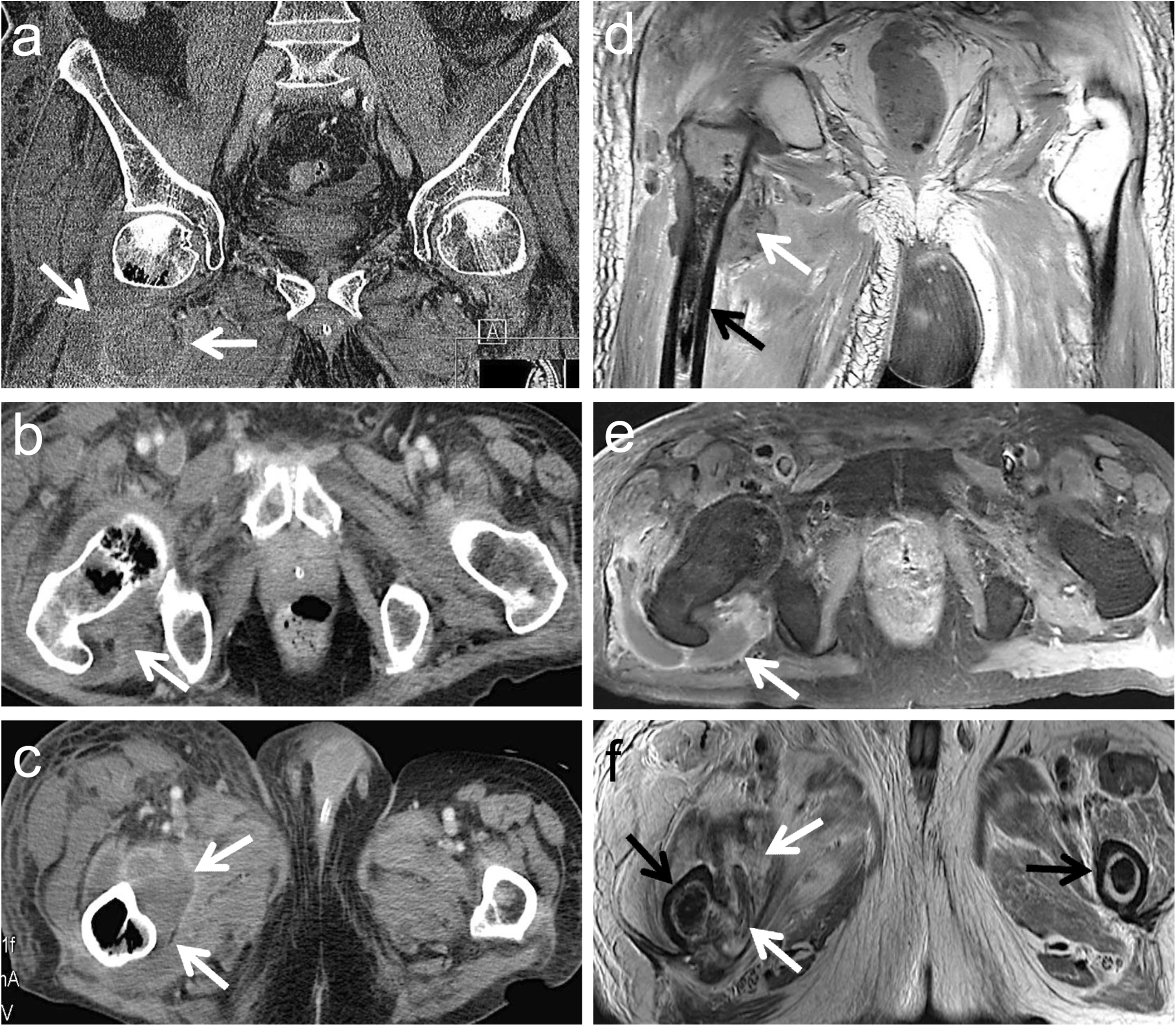

Supplement: Supplementary file 1 — Authors’ original file for figure 1 [file 12879_2014_3791_MOESM1_ESM.tif]

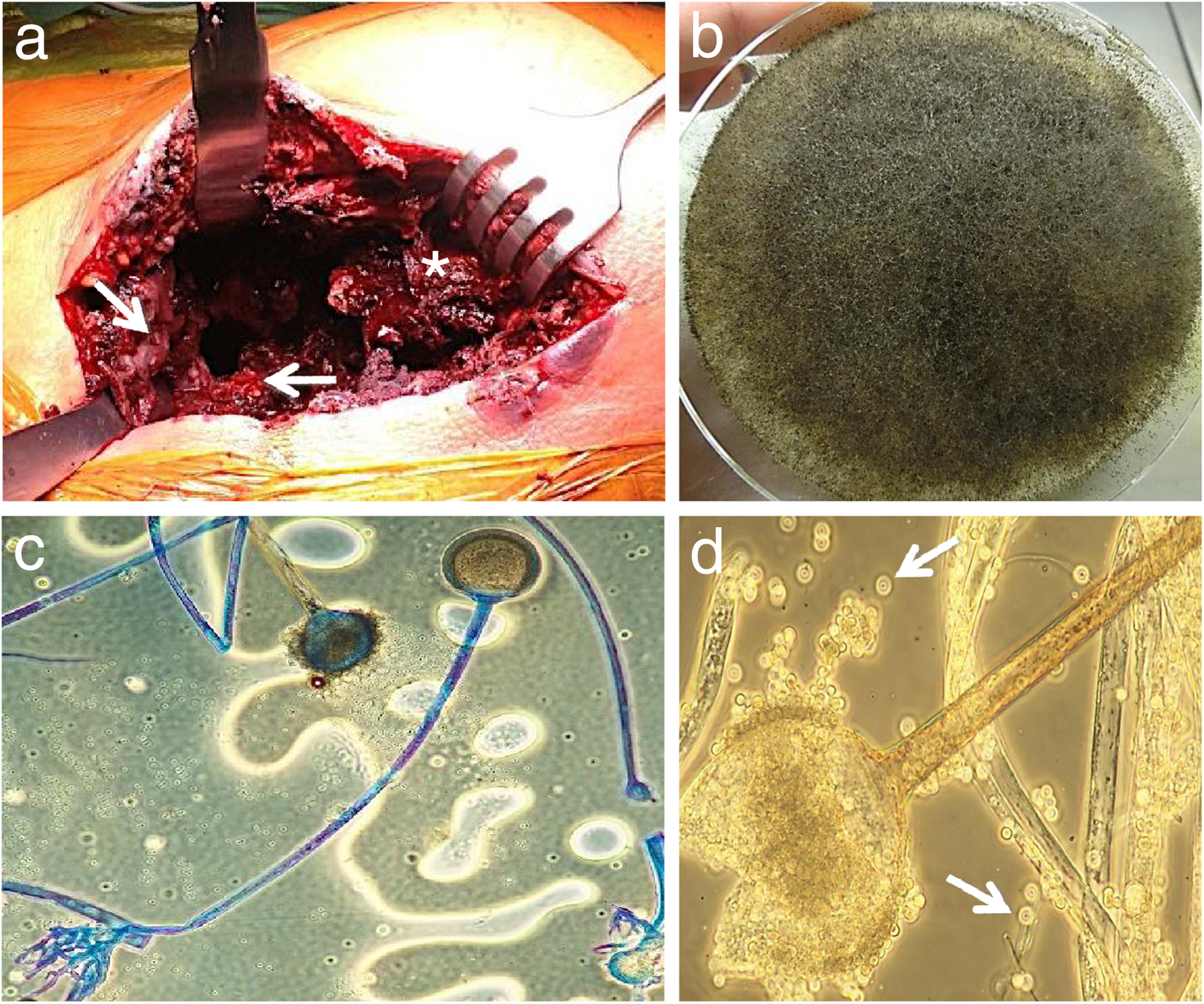

Supplement: Supplementary file 2 — Authors’ original file for figure 2 [file 12879_2014_3791_MOESM2_ESM.tif]
